# Supplementary material for: Residential energy use emissions dominate health impacts from exposure to ambient particulate matter in India
Source: Nat Commun. 2018 Feb 12;9:617. doi: 10.1038/s41467-018-02986-7 (PMC5809377; doi:10.1038/s41467-018-02986-7)
Supplement: Supplementary file 1 — Supplementary Information [file 41467_2018_2986_MOESM1_ESM.pdf]

Supplementary information: Residential energy use emissions dominate health impacts from exposure to ambient particulate matter in India

Luke Conibear*[,1,2], Edward W. Butt[2], Christoph Knote[3], Stephen R. Arnold[2] and Dominick V. Spracklen[2]

[1] Engineering and Physical Sciences Research Council (EPSRC) Centre for Doctoral Training (CDT) in Bioenergy, University of Leeds, Leeds, LS2 9JT, UK

[2] Institute for Climate and Atmospheric Science, School of Earth and Environment, University of Leeds, Leeds, LS2 9JT, UK

[3] Meteorological Institute, Ludwig-Maximilians-University Munich, Theresienstr. 37, 80333, Munich, Germany

Correspondence and material requests addressed to L.C (email: pmlac@leeds.ac.uk)

Supplementary Methods

Model performance for the control scenario will be evaluated using statistical metrics for evaluating air quality models[1], including mean bias (MB), normalized mean bias (NMB), root mean square error (RMSE), normalized mean absolute error (NMAE) and Pearson's correlation coefficient (r). These have been used in previous studies for evaluating regional, air quality models[2–5]. The MB indicates the level of overestimation (positive values) or underestimation (negative values) by the model (Equation 1). N represents the total number of model-observation pair values while M_i and O_i represent the ith model and observed values, respectively. The NMB represents the model bias relative to the observations without being overly influenced by small numbers in the denominator (Equation 2). The RMSE captures the average error produced by the model (Equation 3). The NMAE represents the mean absolute difference between model and observations relative to the observations (Equation 4). The extent of the linear relationship between model and observations is given by the Pearson's correlation coefficient (Equation 5). The over bars represent the respective mean. MB has the same

units as the variable being evaluated, while all other metrics are unit-less. The gradient of best fit is determined through the Python package SciPy using least-squares solution to a linear matrix equation.

$$MB = \frac{1}{N}\sum_{i=0}^{N}\left(M_i - O_i\right) \quad (1)$$

$$NMB = \frac{\sum_{i=1}^{N}\left(M_i - O_i\right)}{\sum_{i=1}^{N} O_i} \quad (2)$$

$$RMSE = \sqrt{\frac{\sum_{i=1}^{N}\left(M_i - O_i\right)^2}{N}} \quad (3)$$

$$NMAE = \frac{\sum_{i=1}^{N}\left|M_i - O_i\right|}{\sum_{i=1}^{N} O_i} \quad (4)$$

$$r = \frac{\sum_{i=0}^{N}\left(M_i - M\right)\left(O_i - O\right)}{\sqrt{\sum_{i=1}^{N}\left(M_i - \overline{M}\right)^2 \sum_{i=0}^{N}\left(O_i - \overline{O}\right)^2}} \quad (5)$$

Model performance benchmarks in simulating meteorology for air quality for temperature are < ±0.5 K for MB and < 2 K for NMAE, while for wind speed are <±0.5 m s^{-1} for MB and <2 m s^{-1} for RMSE[2].

Satellite aerosol observation allows for high horizontal resolutions, though is restricted to daytime measurements, has no vertical resolution and can lack in accuracy relative to ground based measurements[6,7]. Data from MODIS Aqua was used due to Terra experiencing a calibration issue in global land AOD for collection 5[8]. Collection 6 (C6) was used for model evaluation as it is known to have statistically significant improvement in aerosol retrieval algorithms over some urban areas[9], and non-linearities between the collections (6 and 5) arise due to pixel selection and calibration[10]. There are uncertainties associated with cloud contamination, surface overlaps and daylight background noise[11]. MODIS has been found to overestimate AOD over land surfaces[12,13] specifically over the IGP[14], underestimates AOD over semi-arid areas with high dust concentrations[8] and does not retrieve

aerosol information at night or over bright surfaces such as the Thar desert[15–17]. The Indian summer is known to be cloudy as this could affect the AOD retrievals and sampling frequency[18]. Background reflection can be very high when aerosol layers are above optically thick clouds, hence the large sensitivity of aerosol absorption to vertical cloud and particle distributions[19]. MODIS AOD has been proved to compare well with in-situ observations over India[20,21].

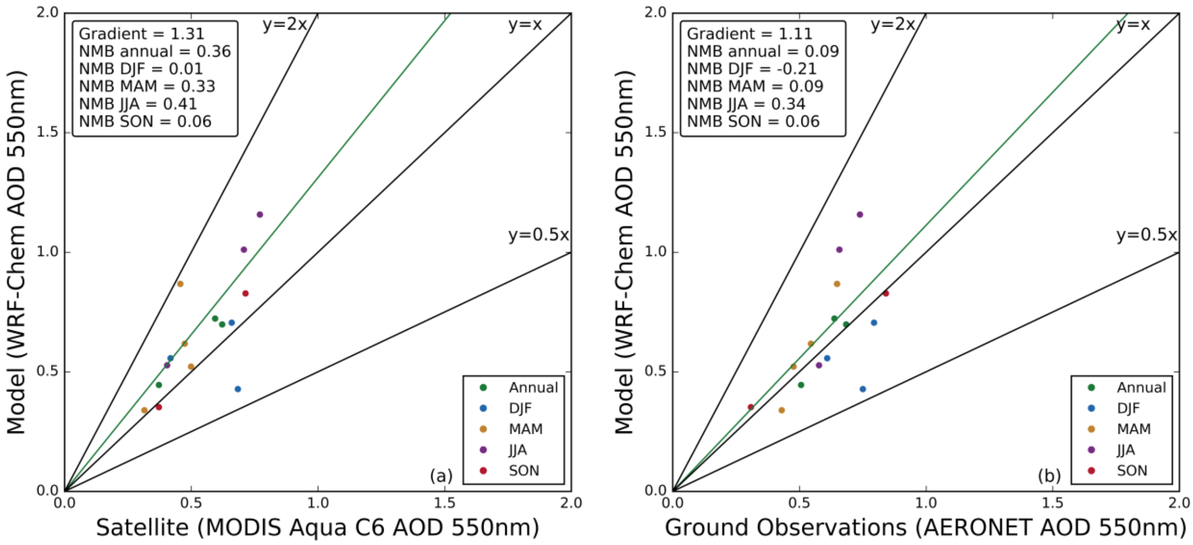

Supplementary Figure 1: Evaluation of annual and seasonal mean model AOD at 550 nm. *(a) comparison of model and satellite (MODIS Aqua C6) at AERONET locations. (b) comparison of model and measured (AERONET). The best-fit line (green), 1:1, 2:1 and 1:2 lines are shown (black). NMB and slope of best-fit line are given inset.*

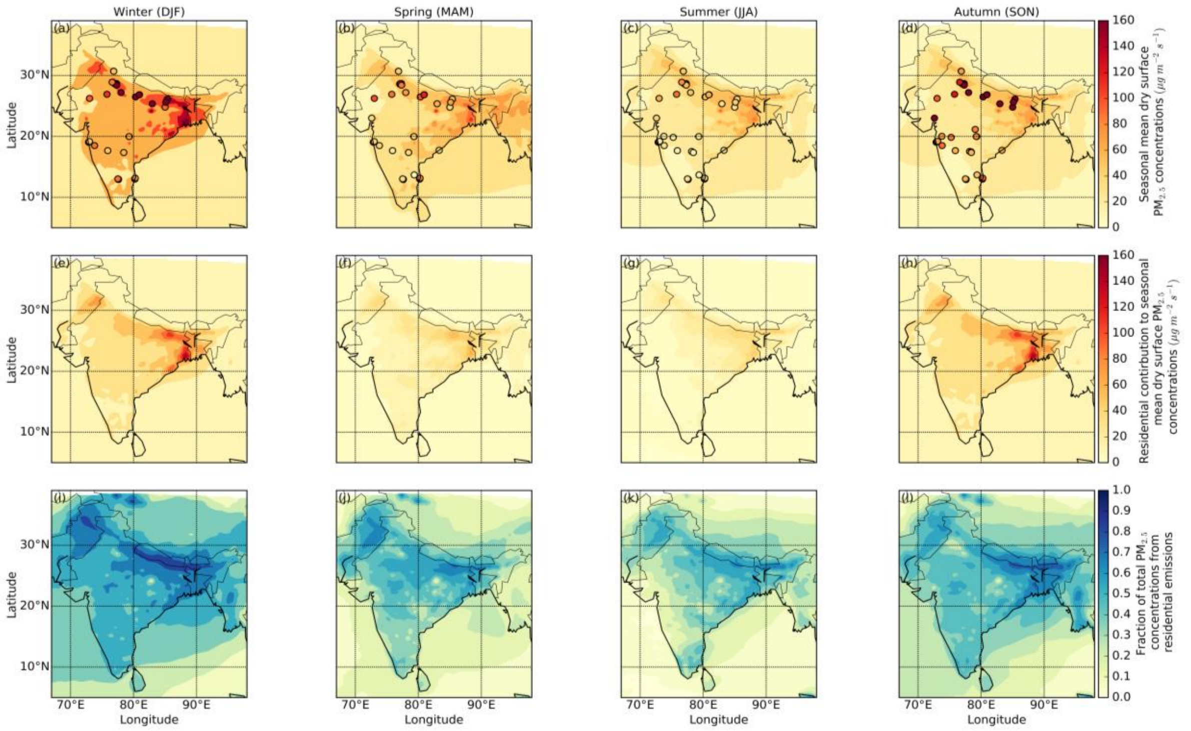

Supplementary Figure 2: Seasonal mean $PM_{2.5}$ concentrations for 2014. *(a) to (d) model results (background) for 2014 for all sources are compared with ground-measurements (filled circles) from 2016, winter to autumn. (e) to (h) Residential sector $PM_{2.5}$, winter to summer. (i) to (l) Fraction from the residential sector, winter to summer.*

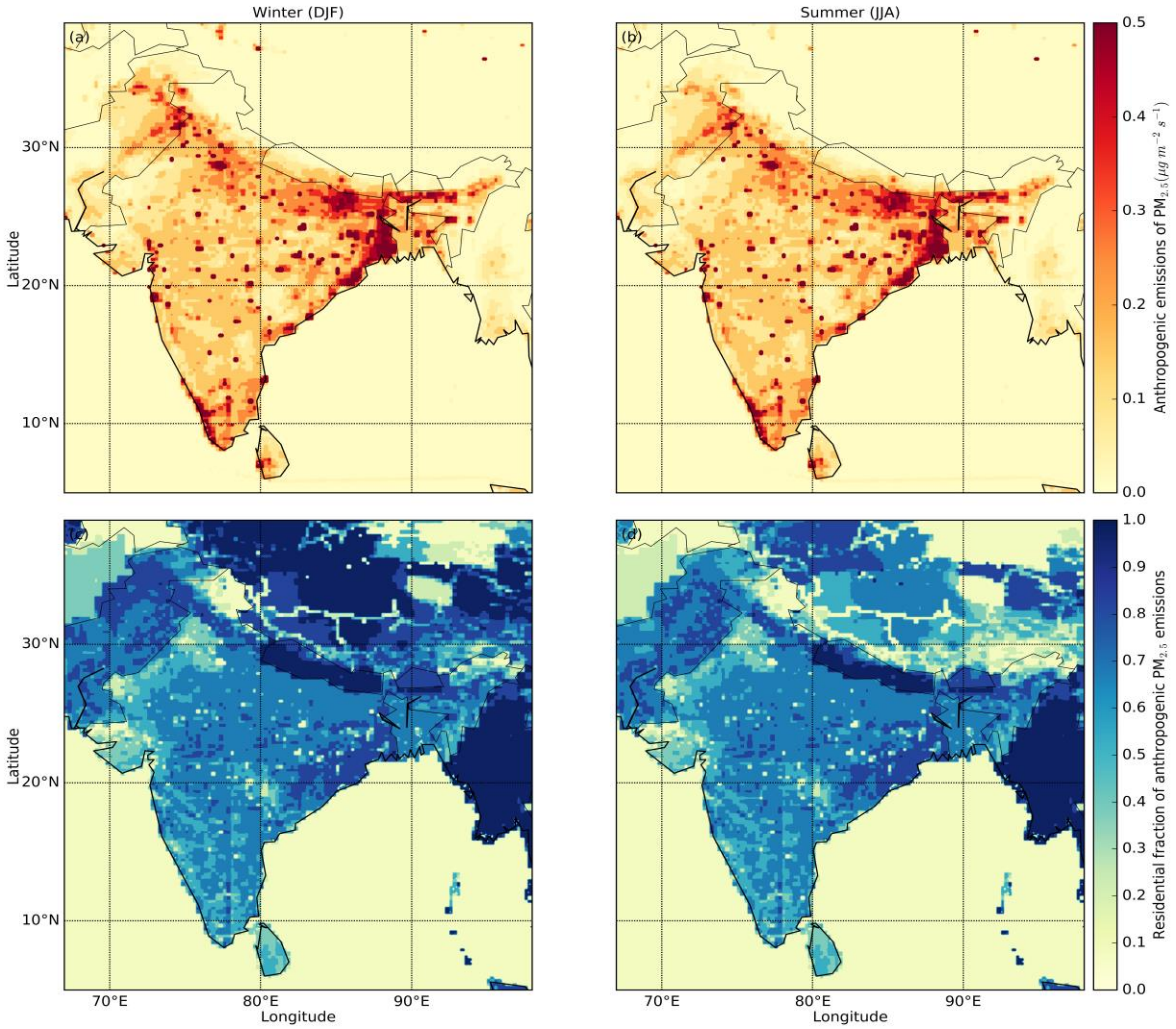

Supplementary Figure 3: Seasonal mean anthropogenic $PM_{2.5}$ emissions. *Anthropogenic $PM_{2.5}$ emissions in (a) winter and (b) summer. Fractional contributions from the residential sector for (c) winter and (d) summer.*

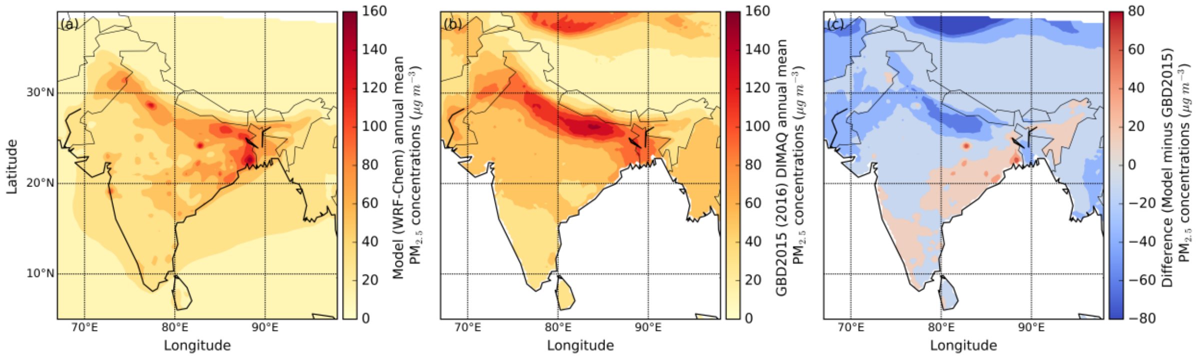

Supplementary Figure 4: Comparison of annual mean $PM_{2.5}$ to GBD2015. *(a) Model (WRF-Chem). (b) GBD2015 (2016)*[22] *DIMAQ. (c) Model minus GBD2015.*

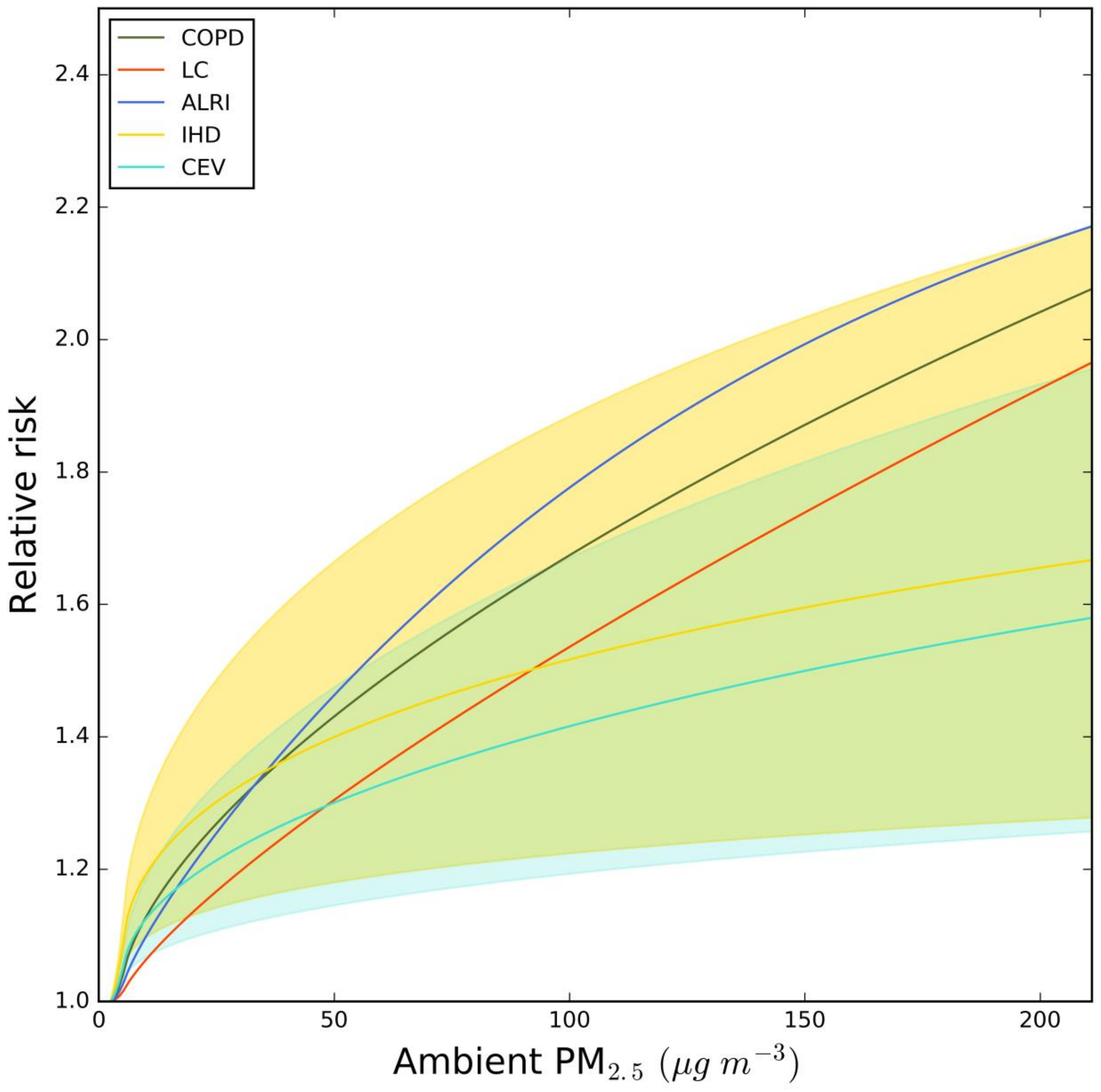

Supplementary Figure 5: Relative risk (RR) as a function of annual-average ambient PM$_{2.5}$ concentrations for different diseases from GBD2015. *Mean exposure-response shown in bold line for IHD and CEV, with shaded regions representing the variation with age groups.*

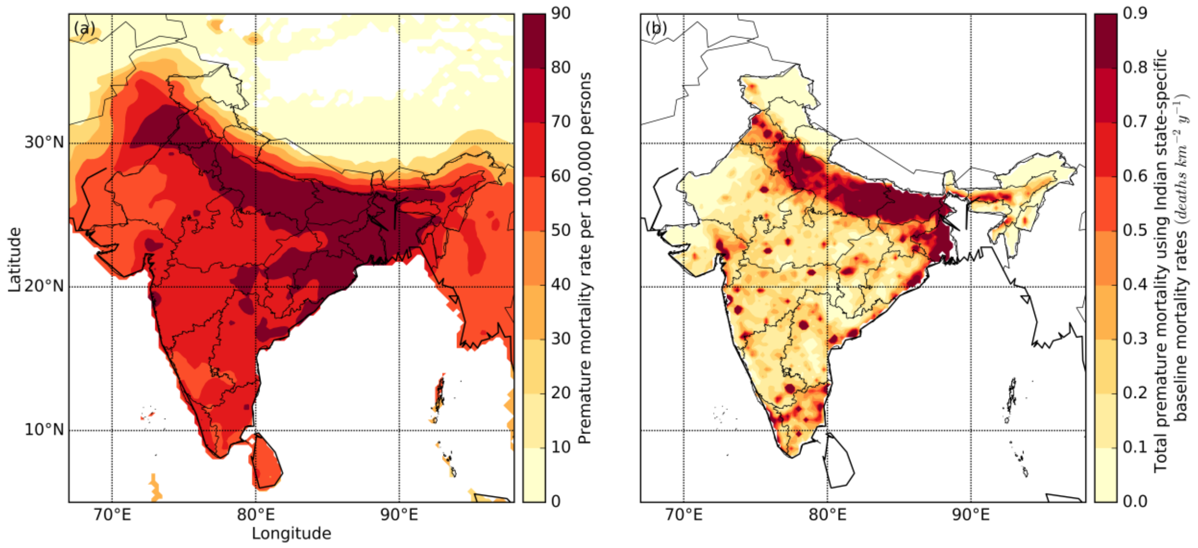

Supplementary Figure 6: Premature mortality estimates from exposure to ambient $PM_{2.5}$ in India. *(a) Premature mortality rate per 100,000 persons. (b) Premature mortality estimate using Indian state-specific baseline mortality rates*[23]*, where white space represents where there was no state-specific baseline mortality rate data available.*

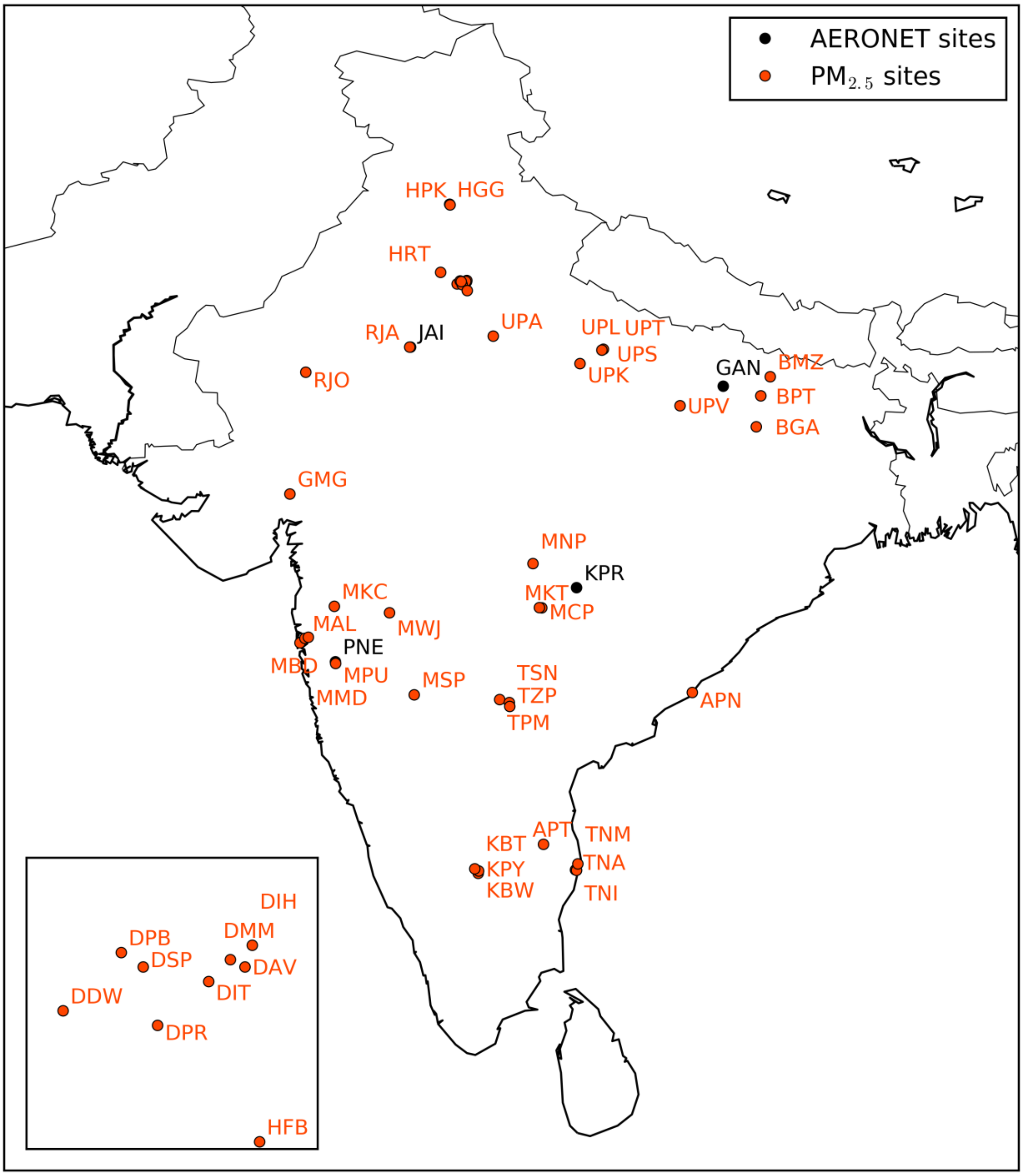

Supplementary Figure 7: Model domain showing ground measurement sites for aerosol optical depth (AOD) from AERONET and $PM_{2.5}$. *The Delhi region is expanded in the bottom left.*

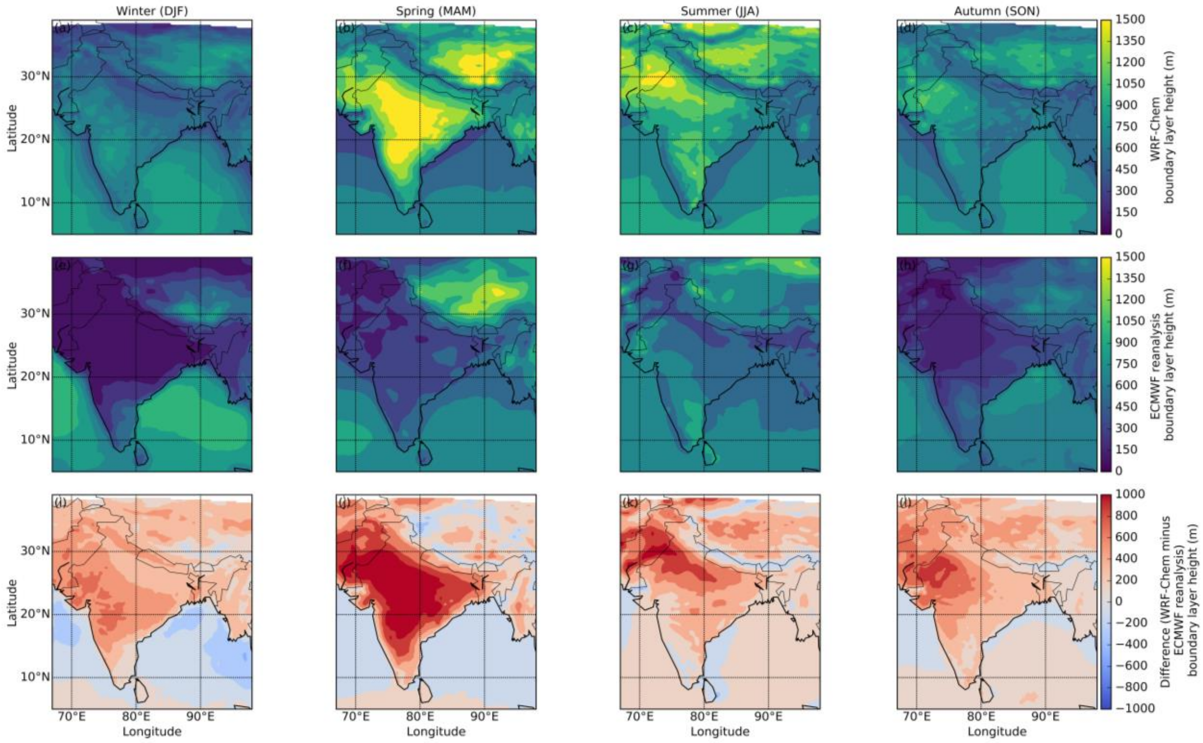

Supplementary Figure 8: Spatial distribution of seasonal mean boundary layer height for 2014. *(a) to (d) WRF-Chem. (e) to (h) ECMWF global reanalyses. (i) to (l) Difference (WRF-Chem minus ECMWF). Results shown for Winter through Autumn, see labels at top of figure.*

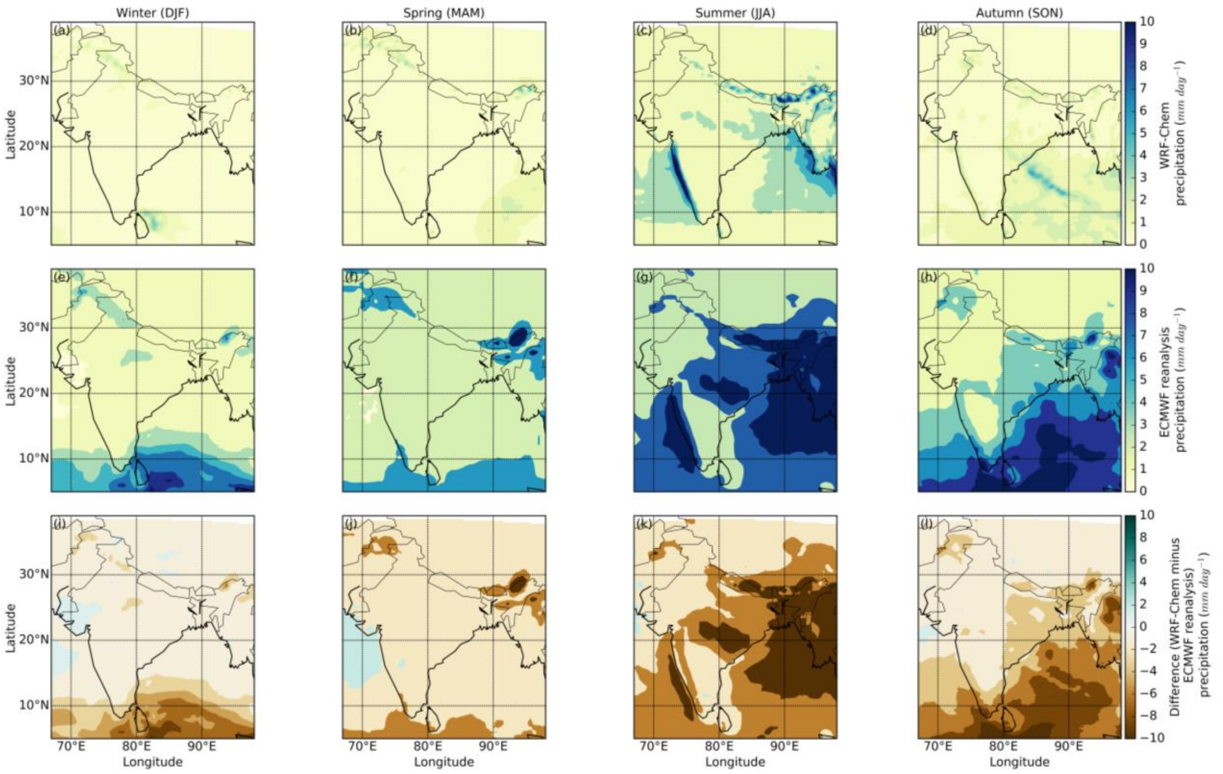

Supplementary Figure 9: Spatial distribution of seasonal mean total precipitation for 2014. *(a) to (d) WRF-Chem. (e) to (h) ECMWF global reanalyses. (i) to (l) Difference (WRF-Chem minus ECMWF). Results shown for Winter through Autumn, see labels at top of figure.*

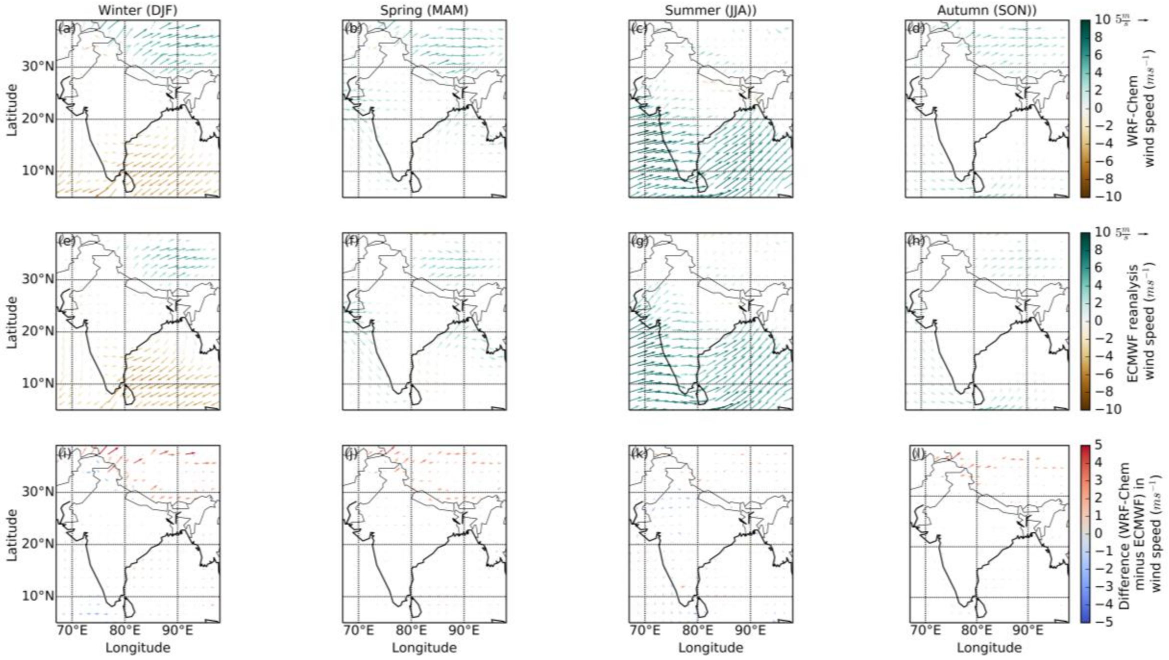

Supplementary Figure 10: Spatial distribution of seasonal mean wind speed and direction for 2014. *(a) to (d) WRF-Chem. (e) to (h) ECMWF global reanalyses. (i) to (l) Difference (WRF-Chem minus ECMWF). Results shown for Winter through Autumn, see labels at top of figure.*

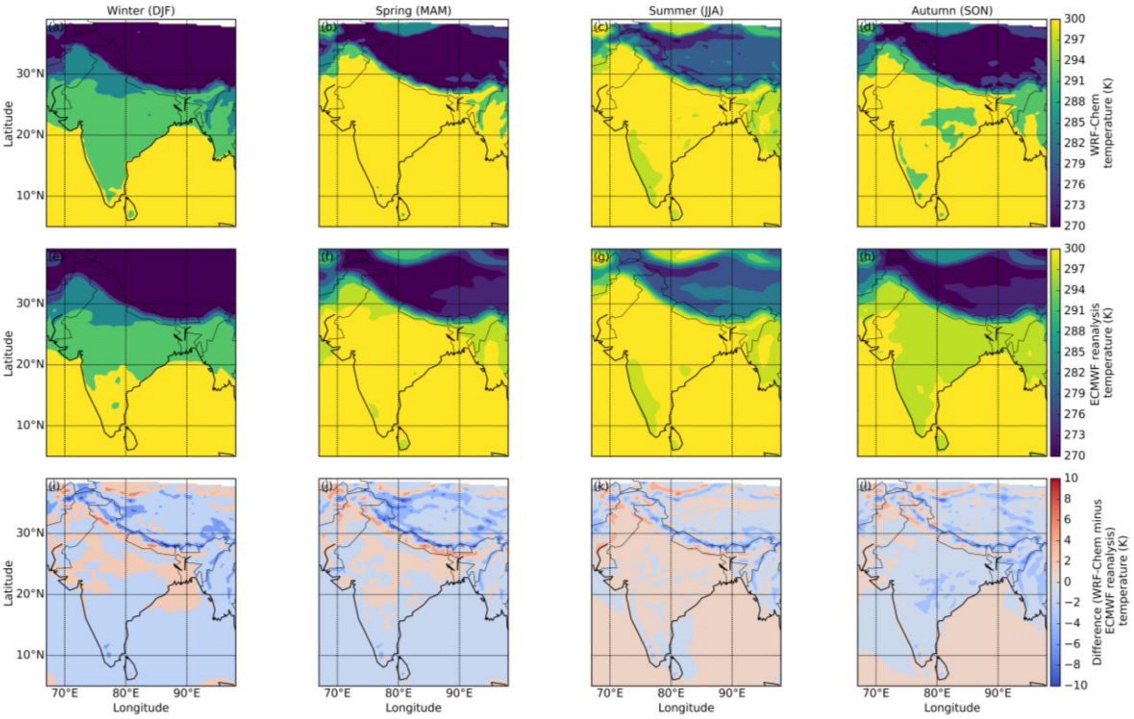

Supplementary Figure 11: Spatial distribution of seasonal mean temperature for 2014. *(a) to (d) WRF-Chem. (e) to (h) ECMWF global reanalyses. (i) to (l) Difference (WRF-Chem minus ECMWF). Results shown for Winter through Autumn, see labels at top of figure.*

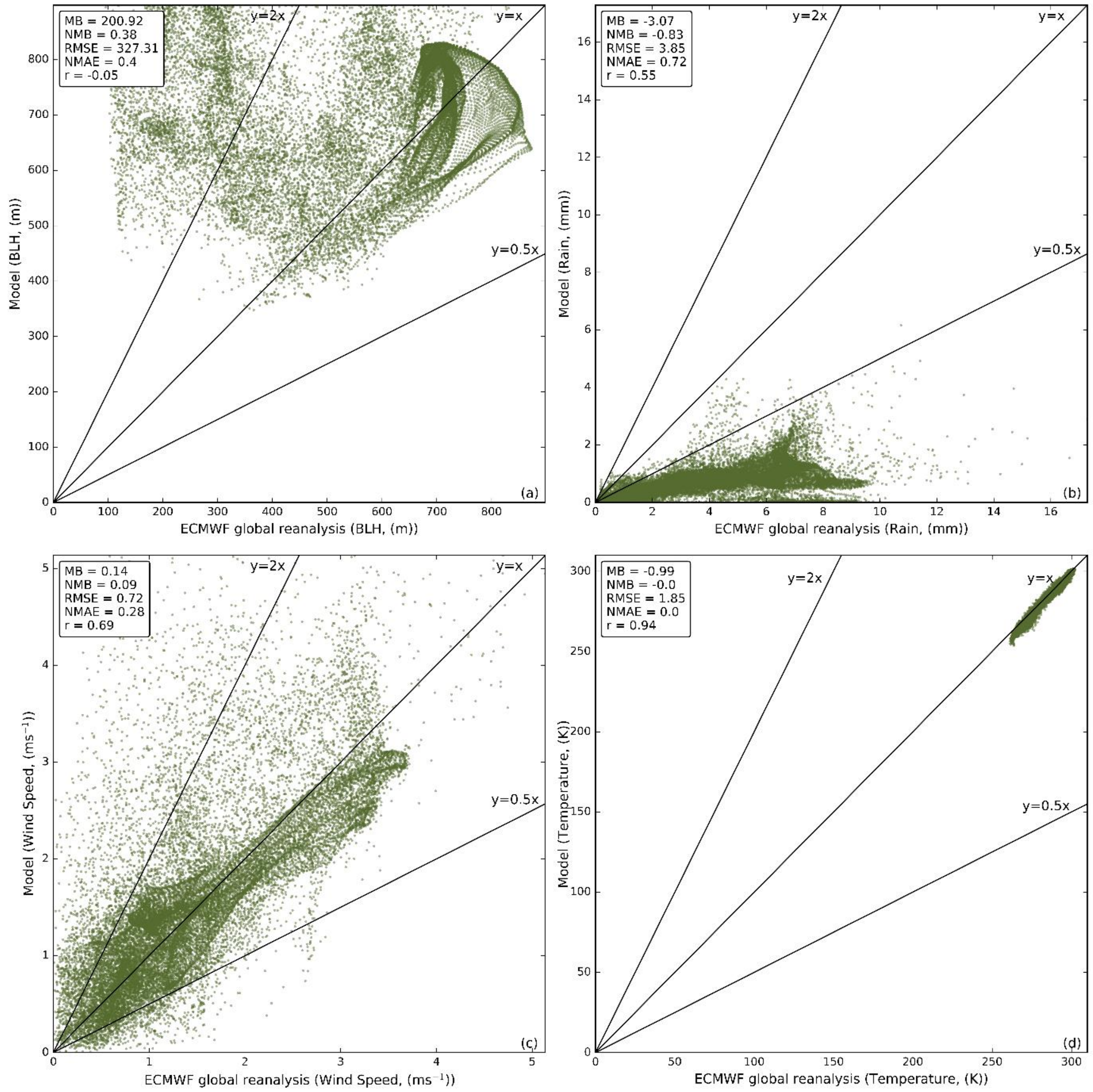

Supplementary Figure 12: Annual mean meteorology correlations between model and ECMWF global reanalyses at each grid cell. *(a) boundary layer height, (b) total precipitation, (c) wind speed and (d) temperature for 2014.*

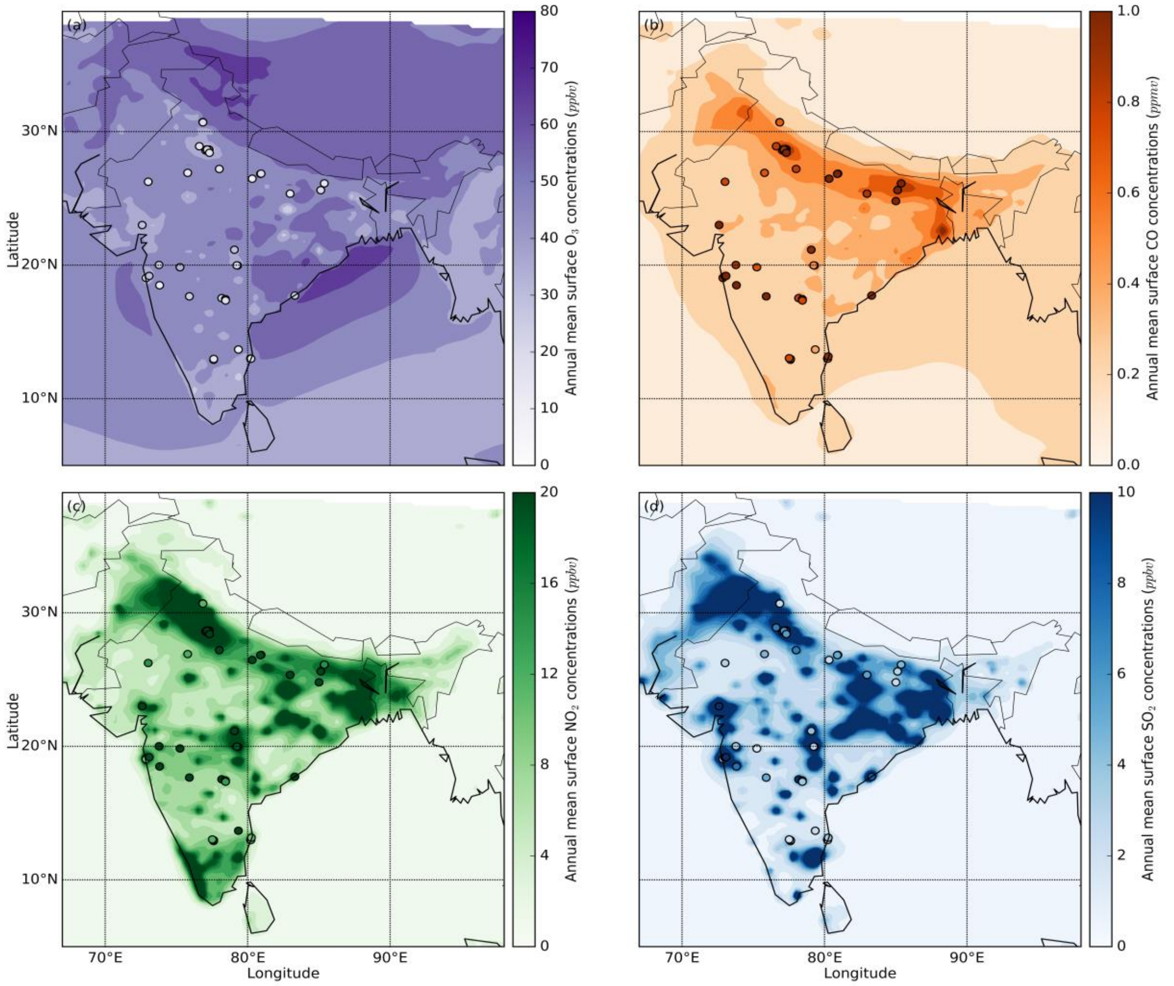

Supplementary Figure 13: Comparison of observed and simulated annual mean surface level gas-phase concentrations. *(a) O_3. (b) CO. (c) NO_2. (d) SO_2.*

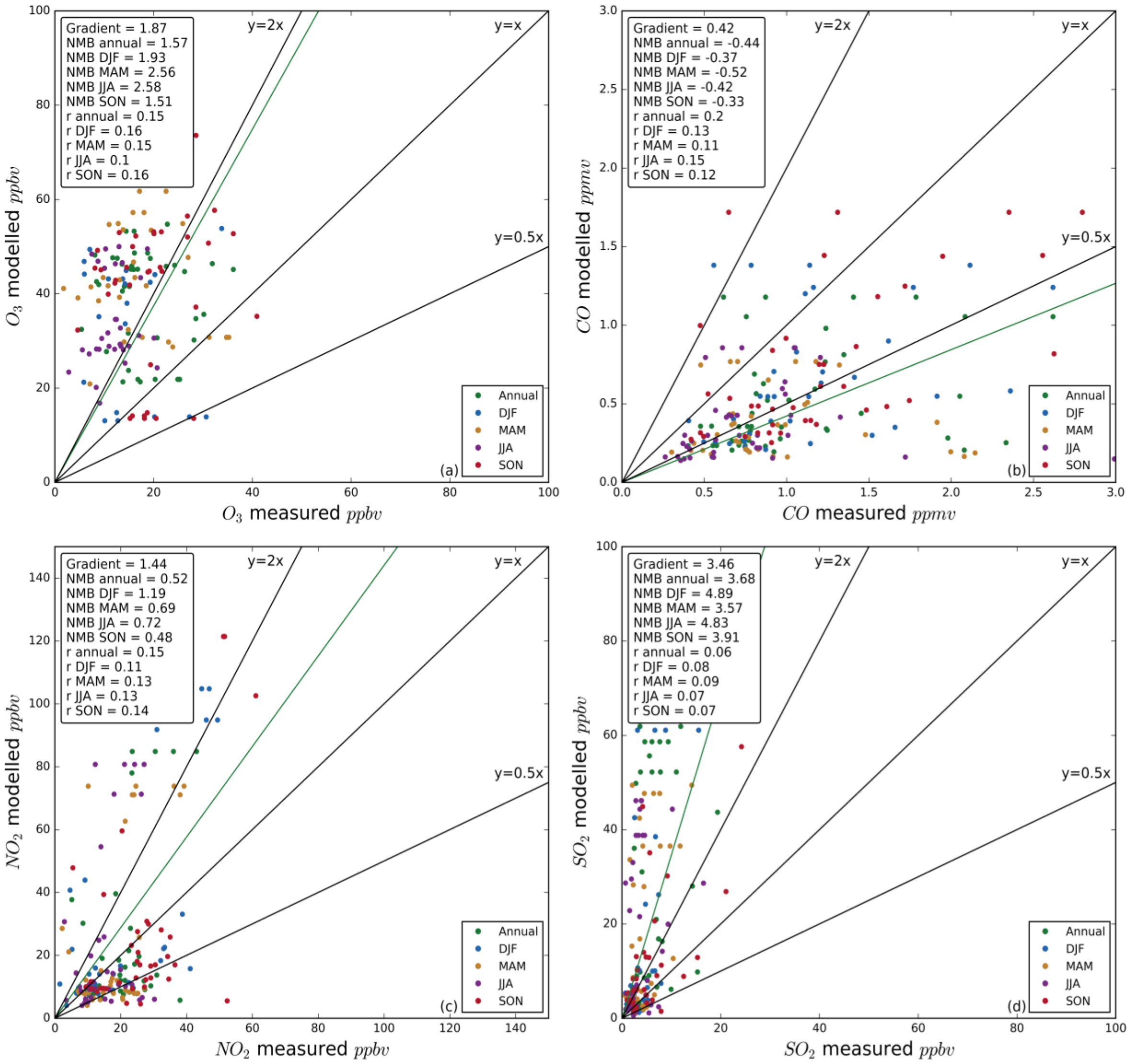

Supplementary Figure 14: Evaluation of annual mean surface level gas-phase concentrations for 2014 for model and ground measurements. *(a) O_3, (b) CO, (c) NO_2 and (d) SO_2. Error bars show one standard deviation.*

Supplementary Table 1: Estimated premature mortality associated with ambient $PM_{2.5}$ exposure in India per disease from both subtraction and attribution methods. *Values in parentheses represent the 95% uncertainty intervals (95UI). Sectors are agriculture (AGR), biomass burning (BBU), dust (DUS), power generation (ENE), industrial non-power (IND), residential energy use (RES) and land transport (TRA).*

			AGR	BBU	DUS	ENE	IND	RES	TRA
**Premature mortality reduction per year**	**Subtraction method**	**COPD**	0	4	0	33	24	92	16
		**($x10^3$)**	(0-1)	(3-7)	(0-0)	(21-49)	(16-36)	(56-135)	(10-24)
		**IHD**	0	3	0	22	16	64	11
		**($x10^3$)**	(0-0)	(3-4)	(0-0)	(19-28)	(14-20)	(53-79)	(9-13)
		**LC**	0	0	0	3	2	8	1
		**($x10^3$)**	(0-0)	(0-0)	(0-0)	(2-4)	(1-3)	(5-10)	(1-2)
		**CEV**	0	2	0	12	9	34	6
		**($x10^3$)**	(0-0)	(1-2)	(0-0)	(9-15)	(7-11)	(25-42)	(4-7)
		**ALRI**	0	3	0	21	15	58	10
		**($x10^3$)**	(0-0)	(1-4)	(0-0)	(9-26)	(7-19)	(23-74)	(4-12)
**Premature mortality reduction per year**	**Attribution method**	**COPD**	1	9	0	65	51	161	32
		**($x10^3$)**	(1-2)	(6-13)	(0-0)	(43-97)	(33-75)	(105-238)	(21-48)
		**IHD**	1	10	0	73	57	180	36
		**($x10^3$)**	(1-2)	(7-13)	(0-0)	(55-96)	(43-74)	(135-236)	(27-47)
		**LC**	0	1	0	4	3	10	2
		**($x10^3$)**	(0-0)	(0-1)	(0-0)	(3-6)	(2-4)	(7-14)	(1-3)
		**CEV**	0	4	0	30	23	73	15
		**($x10^3$)**	(0-1)	(3-5)	(0-0)	(22-38)	(17-30)	(55-94)	(11-19)
		**ALRI**	1	5	0	35	27	87	17
		**($x10^3$)**	(0-1)	(2-6)	(0-0)	(15-47)	(12-36)	(38-12)	(8-23)

Supplementary Table 2: Estimated years of life lost (YLL) associated with ambient $PM_{2.5}$ exposure in India per sector from both subtraction and attribution methods. *Values in parentheses represent the 95% uncertainty intervals (95UI). Sectors are agriculture (AGR), biomass burning (BBU), dust (DUS), power generation (ENE), industrial non-power (IND), residential energy use (RES) and land transport (TRA).*

	AGR	BBU	DUS	ENE	IND	RES	TRA
**YLL Subtraction ($x10^3$)**	26 (17-34)	316 (188-401)	-8 (-4--10)	2,313 (1,324-2,956)	1,699 (997-2,177)	6,553 (3,573-8,296)	1,100 (645-1,408)
**YLL Attribution ($x10^3$)**	86 (51-114)	688 (407-915)	-16 (-10--22)	5,162 (3,056-6,860)	4,001 (2,368-5,316)	12,690 (7,513-16,864)	2,538 (1,503-3,373)

Supplementary Table 3: Model Setup and parameterisation used in the WRF-Chem model.

**Model Setup and Parameterisation**	
**Process**	**Method**
**Domain**	60° to 100° East, 0° to 40° North
**Timestep**	180 seconds, with Runge-Kutta 2nd and 3rd order time integration
**Horizontal**	Resolution of 30 km along a 140x140 grid, with Arakawa C-grid staggering and 2nd to 6th order advection schemes
**Vertical**	27 vertical levels (top at 10 hPa) with terrain-following hydrostatic pressure coordinates and 2nd to 6th order advection schemes
**Precipitation microphysics**	Thompson scheme[24]
**Longwave radiation**	RRTM longwave[25], called every 30 mins
**Shortwave radiation**	RRTM shortwave[26], called every 30 mins
**Boundary layer physics**	Mellor-Yamada Nakanishi and Niino 2.5[27], called every timestep
**Land surface**	Noah Land Surface Model[28]
**Convective parameterisation**	Grell 3-D ensemble[29], called every 60 seconds
**Gas-phase chemistry scheme**	MOZART-4 using KPP, chem_opt=201[30], called every 12 mins
**Photolysis scheme**	Madronich FTUV[31], called every 30 mins
**Aerosol scheme**	MOSAIC 4-bin[32], called every 12 mins
**Dust**	GOCART online with AFWA, dust_opt=3[33,34]
**Initial & boundary chemistry/aerosol**	MOZART-4/GEOS5[35]
**Initial & boundary meteorology**	NCEP GFS and NCEP FNL[36,37]

Supplementary Table 4: Ground measurement air quality stations.

State	City	Station	Code	Lat	Lon
Andhra	Tirupati	Tirumala	APT	79.35	13.68
Pradesh	Visakhapatnam	GVMC Ram Nagar	APN	83.31	17.72
	Gaya	Gaya Collectorate	BGA	85.01	24.79
Bihar	Muzaffarpur	Muzaffarpur Collectorate	BMZ	85.38	26.12
	Patna	IGSC Planetarium Complex	BPT	85.13	25.61
		Anand Vihar	DAV	77.30	28.65
		Dwarka	DDW	77.05	28.59
		IHBAS	DIH	77.31	28.68
		Income Tax Office	DIT	77.25	28.63
Delhi	Delhi	Mandir Marg	DMM	77.28	28.66
		Punjabi Bagh	DPB	77.13	28.67
		R K Puram	DPR	77.18	28.57
		Shadipur	DSP	77.16	28.65
Gurjarat	Ahmedabad	Manianagar	GMG	72.60	23.00
	Faridabad	Sector16A Faridabad	HFB	77.32	28.41
	Gurgaon	HSPCB Gurgaon	HGG	76.85	30.71
Haryana	Panchkula	Panchkula	HPK	76.86	30.69
	Rohtak	Rohtak	HRT	76.61	28.90
		BTM	KBT	77.61	12.91
Karnataka	Bengalaru	BWSSB	KBW	77.62	12.97
		Peenya	KPY	77.52	13.03
	Aurangabad	More Chowk-Waluj	MWJ	75.25	19.84
	Chandrapur	Chandrapur	MCP	79.30	19.97
		MIDC Khutala	MKT	79.24	19.98
Maharashtra	Mumbai	MPCB Bandra	MBD	72.87	19.04
		NMMC Airoli	MAL	73.00	19.16
	Nagpur	Civil Lines	MNP	79.07	21.15
	Nashik	KTHM College	MKC	73.78	20.01

	Pune	Karve Road Pune	MPU	73.82	18.46
	Solapur	Solapur	MSP	75.91	17.66
	Thane	Pimpleshwar Mandir	MMD	73.09	19.19
Rajasthan	Jaipur	Jaipur	RJA	75.79	26.91
	Jodhpur	Jodhpur	RJO	73.02	26.24
Tamil Nadu	Chennai	Alandur	TNA	80.20	13.00
		IIT	TNI	80.23	12.99
		Manali	TNM	80.26	13.16
Telangana	Hyderabad	IDA Pashamylaram	TPM	78.18	17.53
		Sanathnagar	TSN	78.44	17.45
		Zoo Park	TZP	78.45	17.35
Uttar Pradesh	Agra	Sanjay Palace	UPA	78.01	27.20
	Kanpur	Nehru Nagar	UPK	80.32	26.47
	Lucknow	Central School	UPS	80.90	26.83
		Lalbagh	UPL	80.94	26.85
		Talkatora	UPT	80.90	26.83
	Varanasi	Ardhali Bazar	UPZ	82.98	25.35

Supplementary Table 5: AERONET stations.

**State**	**City**	**Station**	**Code**	**Lat**	**Lon**
Rajasthan	Jaipur	Jaipur	JAI	26.90	75.80
Uttar Pradesh	Kanpur	Kanpur	KPR	26.51	80.23
Maharashtra	Pune	Pune	PNE	18.52	73.85
Jharkhand	Bokaro	Gandhi College	GAN	25.87	84.13

Supplementary References

1. Yu, S., Eder, B., Dennis, R., Chu, S.-H. & Schwartz, S. E. New unbiased symmetric metrics for evaluation of air quality models. *Atmos. Sci. Lett.* **7,** 26–34 (2006).
2. Emery, C., Tai, E. & Yarwood, G. Enhanced Meteorological Modeling and Performance Evaluation for Two Texas Ozone Episodes. *Air Sci.* 1–235 (2001).
3. Kumar, R. *et al.* Simulations over South Asia using the Weather Research and Forecasting model with Chemistry (WRF-Chem): chemistry evaluation and initial results. *Geosci. Model Dev.* **5,** 619–648 (2012).
4. Kumar, R., Naja, M., Pfister, G. G., Barth, M. C. & Brasseur, G. P. Simulations over South Asia using the Weather Research and Forecasting model with Chemistry (WRF-Chem): Set-up and meteorological evaluation. *Geosci. Model Dev.* **5,** 321–343 (2012).
5. Zhang, M. *et al.* Evaluation of the Models-3 Community Multi-scale Air Quality (CMAQ) modeling system with observations obtained during the TRACE-P experiment: Comparison of ozone and its related species. *Atmos. Environ.* **40,** 4874–4882 (2006).
6. Ramachandran, S., Kedia, S. & Sheel, V. Spatiotemporal characteristics of aerosols in India: Observations and model simulations. *Atmos. Environ.* **116,** 225–244 (2015).
7. Chung, C. E., Ramanathan, V., Kim, D. & Podgorny, I. A. Global anthropogenic aerosol direct forcing derived from satellite and ground-based observations. *J. Geophys. Res.* **110,** 1–17 (2005).
8. Levy, R. C. *et al.* Global evaluation of the Collection 5 MODIS dark-target aerosol products over land. *Atmos. Chem. Phys.* **10,** 10399–10420 (2010).
9. Ford, B. & Heald, C. L. Exploring the uncertainty associated with satellite-based estimates of premature mortality due to exposure to fine particulate matter. *Atmos. Chem. Phys.* **156,** 3499–3523 (2016).
10. Levy, R. C. *et al.* The Collection 6 MODIS aerosol products over land and ocean. *Atmos. Meas. Tech.* **6,** 2989–3034 (2013).
11. Feng, Y., Kotamarthi, V. R., Coulter, R., Zhao, C. & Cadeddu, M. Radiative and thermodynamic responses to aerosol extinction profiles during the pre-monsoon month over South Asia. *Atmos.*

Chem. Phys. **16,** 247–264 (2016).

12. Jethva, H., Satheesh, S. K. & Srinivasan, J. Evaluation of Moderate-Resolution Imaging Spectroradiometer (MODIS) Collection 004 (C004) aerosol retrievals at Kanpur, Indo-Gangetic Basin. *J. Geophys. Res. Atmos.* **112,** 1–9 (2007).
13. Jethva, H., Satheesh, S. K., Srinivasan, J. & Moorthy, K. K. How good is the assumption about visible surface reflectance in MODIS aerosol retrieval over Land? A comparison with aircraft measurements over an urban site in India. *IEEE Trans. Geosci. Remote Sens.* **47,** 1990–1998 (2009).
14. Bibi, H. *et al.* Intercomparison of MODIS, MISR, OMI, and CALIPSO aerosol optical depth retrievals for four locations on the Indo-Gangetic plains and validation against AERONET data. *Atmos. Environ.* **111,** 113–126 (2015).
15. Levy, R. C. *et al.* A Critical Look at Deriving Monthly Aerosol Optical Depth From Satellite Data. *IEEE Trans. Geosci. Remote Sens.* **47,** 2942–2956 (2009).
16. Remer, L. a. *et al.* Global aerosol climatology from the MODIS satellite sensors. *J. Geophys. Res.* **113,** 1–18 (2008).
17. Kumar, R., Barth, M. C., Pfister, G. G., Naja, M. & Brasseur, G. P. WRF-Chem simulations of a typical pre-monsoon dust storm in northern India: Influences on aerosol optical properties and radiation budget. *Atmos. Chem. Phys.* **14,** 2431–2446 (2014).
18. Cherian, R., Venkataraman, C., Quaas, J. & Ramachandran, S. GCM simulations of anthropogenic aerosol-induced changes in aerosol extinction, atmospheric heating and precipitation over India. *J. Geophys. Res. Atmos.* **118,** 2938–2955 (2013).
19. Seinfeld, J. H. Black carbon and brown clouds. *Geochemistry, Geophys. Geosystems* **1,** 15–16 (2004).
20. Ramachandran, S. Aerosol optical depth and fine mode fraction variations deduced from Moderate Resolution Imaging Spectroradiometer (MODIS) over four urban areas in India. *J. Geophys. Res. Atmos.* **112,** 1–11 (2007).
21. Sharma, D., Singh, D. & Kaskaoutis, D. G. Impact of two intense dust storms on aerosol characteristics and radiative forcing over Patiala, Northwestern India. *Adv. Meteorol.* **2012,** 1–

13 (2012).

22. Shaddick, G. *et al.* Data integration model for air quality: a hierarchical approach to the global estimation of exposures to ambient air pollution. *J. R. Stat. Soc.* 1–23 (2017). doi:10.1111/rssc.12227

23. Chowdhury, S. & Dey, S. Cause-specific premature death from ambient PM2.5 exposure in India: Estimate adjusted for baseline mortality. *Environ. Int.* **91,** 283–290 (2016).

24. Thompson, G., Rasmussen, R. M. & Manning, K. Explicit Forecasts of Winter Precipitation Using an Improved Bulk Microphysics Scheme. Part II: Implementation of a New Snow Parameterization. *Am. Meteorol. Soc.* **136,** 5095–5115 (2008).

25. Mlawer, E. J., Taubman, S. J., Brown, P. D., Iacono, M. J. & Clough, S. A. Radiative transfer for inhomogeneous atmospheres: RRTM, a validated correlated-k model for the longwave. *J. Geophys. Res.* **102,** 16663–16682 (1997).

26. Pincus, R., Barker, H. W. & Morcrette, J.-J. A fast, flexible, approximate technique for computing radiative transfer in inhomogeneous cloud fields. *J. Geophys. Res.* **108,** 1–5 (2003).

27. Nakanishi, M. & Niino, H. An improved Mellor-Yamada Level-3 model: Its numerical stability and application to a regional prediction of advection fog. *Boundary-Layer Meteorol.* **119,** 397–407 (2006).

28. Ek, M. B. *et al.* Implementation of Noah land surface model advances in the National Centers for Environmental Prediction operational mesoscale Eta model. *J. Geophys. Res. Atmos.* **108,** 8851–8867 (2003).

29. Grell, G. A. & Devenyi, D. A generalized approach to parameterizing convection combining ensemble and data assimilation techniques. *Geophys. Res. Lett.* **29,** 10–13 (2002).

30. Emmons, L. K. *et al.* Description and evaluation of the Model for Ozone and Related chemical Tracers, version 4 (MOZART-4). *Geosci. Model Dev. Discuss.* 43–67 (2010). doi:10.5194/gmdd-2-1157-2009

31. Tie, X. *et al.* Effect of clouds on photolysis and oxidants in the troposphere. *J. Geophys. Res.* **108,** 4642, 1–11 (2003).

32. Zaveri, R. A., Easter, R. C., Fast, J. D. & Peters, L. K. Model for Simulating Aerosol Interactions

and Chemistry (MOSAIC). *J. Geophys. Res. Atmos.* **113,** 1–29 (2008).

33. Chin, M. *et al.* Tropospheric Aerosol Optical Thickness from the GOCART Model and Comparisons with Satellite and Sun Photometer Measurements. *J. Atmos. Sci.* **59,** 461–483 (2002).

34. Chin, M., Rood, R. B., Lin, S.-J., Müller, J.-F. & Thompson, A. M. Atmospheric sulfur cycle simulated in the global model GOCART: Model description and global properties. *J. Geophys. Res.* **105,** 24671–24687 (2000).

35. NCAR. ACOM MOZART-4/GEOS-5 global model output. *UCAR* (2016). at <http://www.acom.ucar.edu/wrf-chem/mozart.shtml>

36. NCEP, National Weather Service, NOAA & U.S. Department of Commerce. NCEP Global Forecast System (GFS) Analyses and Forecasts. Research Data Archive at the National Center for Atmospheric Research, Computational and Information Systems Laboratory. (2007). doi:http://rda.ucar.edu/datasets/ds084.6/

37. NCEP, National Weather Service, NOAA & U.S. Department of Commerce. NCEP Final (FNL) Operational Model Global Tropospheric Analyses, continuing from July 1999. Research Data Archive at the National Center for Atmospheric Research, Computational and Information Systems Laboratory. (2000). doi:http://dx.doi.org/10.5065/D6M043C6.
